# Independent validation of a dysphagia dose response model for the selection of head and neck cancer patients to proton therapy

**DOI:** 10.1016/j.phro.2022.09.005

**Published:** 2022-09-17

**Authors:** Petros Kalendralis, Matthijs Sloep, Nibin Moni George, Jasper Snel, Joeri Veugen, Frank Hoebers, Frederik Wesseling, Mirko Unipan, Martijn Veening, Johannes A. Langendijk, Andre Dekker, Johan van Soest, Rianne Fijten

**Affiliations:** a Department of Radiation Oncology (Maastro), GROW School for Oncology and Reproduction, Maastricht University Medical Centre+, Maastricht, the Netherlands; b Department of Radiation Oncology, University of Groningen, University Medical Centre Groningen. Groningen. the Netherlands; c Brightlands Institute for Smart Digital Society (BISS), Faculty of Science and Engineering, Maastricht University, Heerlen, the Netherlands

## Abstract

**Background and purpose:**

The model based approach involves the use of normal tissue complication models for selection of head and neck cancer patients to proton therapy. Our goal was to validate the clinical utility of the related dysphagia model using an independent patient cohort.

**Materials and Methods:**

A dataset of 277 head and neck cancer (pharynx and larynx) patients treated with (chemo)radiotherapy between 2019 and 2021 was acquired. For the evaluation of the model discrimination we used statistical metrics such as the sensitivity, specificity and the area under the receiver operating characteristic curve. After the validation we evaluated if the dysphagia model can be improved using the closed testing procedure, the Brier and the Hosmer-Lemeshow score.

**Results:**

The performance of the original normal tissue complication probability model for dysphagia grade II-IV at 6 months was good (AUC = 0.80). According to the graphical calibration assessment, the original model showed underestimated dysphagia risk predictions. The closed testing procedure indicated that the model had to be updated and selected a revised model with new predictor coefficients as an optimal model. The revised model had also satisfactory discrimination (AUC = 0.83) with improved calibration.

**Conclusion:**

The validation of the normal tissue complication probability model for grade II-IV dysphagia was successful in our independent validation cohort. However, the closed testing procedure indicated that the model should be updated with new coefficients.

## 1 Introduction

Head and neck cancer (HNC) constitutes one of the most common cancer types worldwide. It is estimated that over 400.000 deaths are caused by HNC malignancies annually [1]. In Europe specifically, HNC accounts for 4 % of the cancer incidence with more than 60.000 deaths annually [2]. During the last years, the main goal of several novel photon-based radiotherapy (RT) techniques have been implemented in clinical practice such as intensity-modulated radiation therapy (IMRT) and the Volumetric Modulated Arc Therapy (VMAT). These RT techniques aimed to deliver the optimal radiation dose to the treatment target while minimising the radiation dose to the nearby healthy tissues and organs at risk (OARs) and therefore reducing acute and late radiation-induced toxicities [3]. For instance, dysphagia was one of the main RT-induced complications in HNC patients and can greatly reduce quality of life and cause other late RT induced side effects such as nutritional implications and tube feeding dependence [4].

Protons deliver their maximum amount of energy to a precise depth in the patient (referred to as the Bragg peak) [5]). Therefore, proton therapy (PT) techniques such as intensity-modulated proton therapy (IMPT) can potentially benefit HNC patients treated for palliative or curative purposes [6]. The “model-based approach” (MBA) [7] had as a main goal to initiate a data-driven selection and qualification of patients that will benefit most from PT. It was established by comparing different logistic regression normal tissue complication probability (NTCP) profiles between the most optimal photon and proton RT treatment plans. These insights then enabled clinicians to select those patients for PT that will have a clinical benefit in terms of reduced radiation-induced toxicity rates after the RT treatment, translated in the difference between the proton and photon NTCP profiles estimation (ΔNTCP).The different dose parameters of the different OARs, as well as other clinical variables such as the baseline toxicity scores according to Patient-Reported Outcome (PROMs) questionnaires or physician-rated scores and the tumour location, were included in these NTCP profiles described in the indication protocol for proton therapy (National Indication Protocol for Proton therapy-NIPP) [8].

However, to ensure accurate selection via the MBA, a standardised registration of high quality patient data was required. The ProTRAIT initiative (PROton Therapy ReseArch regIsTry) [9] was established with the goal to systematically register patients data from different tumour groups including demographic data [10] that can support the MBA. Furthermore, the data were transformed in a FAIR (Findable, Accessible, Interoperable, Reusable) data [11] format so that the different NTCP statistical profiles can be validated in a privacy preserving manner using the Personal Health Train (PHT) infrastructure [12]. For instance, the external validation of the MBA-based NTCP models. In this study, we aimed to assess the accuracy and robustness of part of the current NIPP [8], based on the MBA, by using data collected in the ProTRAIT [9]. To this end, we validated the logistic regression-based NTCP model for grade II-IV dysphagia at 6 months (primary setting) as described by the NIPP [8] using data from photon and proton-based RT treatment plans of patients.

## 2 Materials and methods

### 2.1 Developed NTCP model

The NTCP dysphagia model for more than grade two dysphagia in the primary setting is described in the current version of the NIPP [8].The development patient cohort characteristics can be shown in Table S1 of the supplementary material as described by the study of Van den Bosch et al. [13] and the NIPP [8].

### 2.2 External validation cohort

For the external validation of the NTCP logistic regression model, we acquired an independent dataset of 277 patients treated with primary (chemo-)RT in MAASTRO clinic between 2019 and 2021 (70 % males and 30 % females). The Institutional Review Board’s (IRB) approval with number W 19 09 00,063 was acquired for the data acquisition and processing for the purposes of this study. The demographic, clinical and OARs dosimetric characteristics are presented in Table 1. The patients were diagnosed with malignancies of the pharynx and larynx and were treated using photon (263 patients-59 patients received chemotherapy in combination with photon based radiotherapy) and proton-based (14 patients) RT techniques. For the dosimetric characteristics-predictors of the NTCP model of the Table 1 we used the mean/average of the mean radiation dose that was delivered to the organs at risk (OARs) of the oral cavity and the superior, middle and inferior pharyngeal constrictor muscle (PCM) as a measure on central tendency. The average can be defined as the sum of the value of each observation (mean radiation dose) in our dataset divided by the number of observations.

**Table 1:** Patient cohort characteristics (n = 277) that was used for the validation of the NTCP ≥ 2 grade six months dysphagia model.

Treatment modality	N (%)
Photon-based conventional radiotherapy	204 (73)
Proton-based conventional radiotherapy	14(5)
Photon-based chemo-radiotherapy	59(22)

Clinical characteristics	
Clinical T stage 8th edition	N (%)
T1-T2	122(43)
T3-T4	142(51)
Tis	2(1)
Tx	11(4)
Clinical N stage 8th edition	N (%)
≤N2	250(90)
≥N3	18(7)
Nx	9(3)
Tumour location	N (%)
Pharynx	188(68)
Larynx	89(32)

Dosimetric characteristics-predictors of the NTCP model for dysphagia grade ≥ 2 at 6 months (Gy) (The average values of the mean delivered radiation dose)	
Photon-based Dmean oral cavity	33.2(SD = 15.4, variance = 237)
Photon-based Dmean PCM superior	55.5(SD = 17.7,variance = 316)
Photon-based Dmean PCM medium	50.2(SD = 17.4,variance = 305.1)
Photon-based Dmean PCM inferior	38.2(SD = 19.9,variance = 399.5)
Proton-based Dmean oral cavity	24.1(SD = 11.9,variance = 142.4)
Proton-based Dmean PCM superior	35.1(SD = 8.3,variance = 71.1)
Proton-based Dmean PCM medium	41.2(SD = 12.6,variance = 159)
Proton-based Dmean PCM inferior	37.5(SD = 17.9,variance = 323)

Abbreviations: Dmean = Mean radiation dose, PCM = Pharyngeal Constrictor Muscle,

**Fig. 1:**
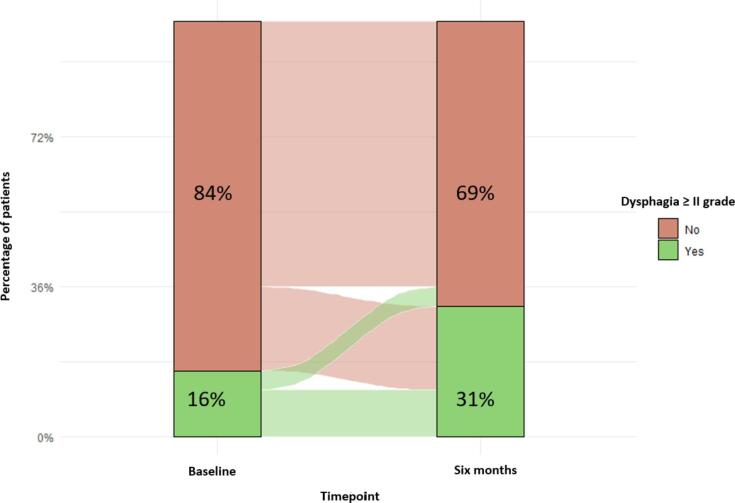
Flowchart that represents the proportion of patients who developed equal or bigger than second-grade dysphagia in the baseline and six months after the end of radiotherapy time points. The percentage of patients who developed second-grade dysphagia six months after radiotherapy was 15% higher compared to the start of the treatment.

The increase in the percentage of patients (15 %) who developed 2nd grade dysphagia in the time period before the start of the RT treatment and after the end of it is one of the important findings presented in Fig. 1.

### 2.3 Statistical analysis

We used the closed testing procedure (CTP) as described and implemented by Vergouwe et al. [14] to validate the dysphagia NTCP model and examine whether the model needs an update. The CTP followed a four levels calibration hierarchy, comparing the updated calibrated models against the original model. Likelihood ratio tests were performed, by testing the statistical significance of the different models indicated by the CTP (ie. p value < 0.05). Following the CTP methodology, we examined four different logistic regression NTCP models. The first one included the calculation of the NTCP values according to the original grade II-IV dysphagia model. For the second model, a new intercept was estimated for the original NTCP model [8] after setting its coefficient equal to 1 (**“**re-calibration in the large''). For the third model, a new updated coefficient of the original NTCP model’s linear predictor was estimated (ie. slope) as well as with the intercept of the model (“Logistic Recalibration”). For the fourth model, we used the complete set of predictor variables used in the original NTCP model, to estimate their respective coefficients (“Model revision/update”). Table S2 of the supplementary material of the study, presents the abovementioned model parameters that have to be estimated. The code used to execute these four aforementioned models was written in the open-source statistical analysis software tool “Comprehensive R Archive Network” [15]. The selected final model was chosen according to the CTP function of Vergouwe et al. [14]**.** The Comprehensive R Archive Network [15] code used for the CTP implementation is publicly available in the Github repository (ProTRAIT/CTP_dysphagia_NTCP.R at main · MaastrichtU-CDS/ProTRAIT (github.com)).The R-based libraries “dplyr” [16], “ModelGood” [17], “ResourceSelection” [18], “rms” [19], “pROC” [20] and “DescTools” [21] were used in the aforementioned code for the discrimination and calibration assessment of the logistic regression models.

### 2.4 Model performance

For model performance, Brier Scores (scale 0 to 1, with the lower values indicating a higher accuracy of the model) were calculated, as suggested by Steyeberg et al. [22]. Moreover, we performed a graphical and quantitative assessment of the calibration of the four different models indicated by the CTP, using the Hosmer–Lemeshow test. This test evaluates the correctness of the predicted compared to the observed probabilities of the NTCP values. The four different models were graphically assessed using the maximum and average difference between the predicted and calibrated probabilities (Emax and Eavg). For the creation of the calibration curves we used the function “calPlot2” from the RStudio [15] package “ModelGood” [17]. For the discrimination evaluation of the four different models, the sensitivity, specificity and the area under the receiver-operating characteristic curve (AUC) were calculated.

## 3 Results

The original grade II-IV dysphagia model presented acceptable discrimination (AUC = 0.80) in the validation dataset, while the “revised model” with new updated coefficients presented excellent discrimination (AUC = 0.83). The receiver operating characteristic (ROC) curves of the four different-CTP indicated-models for grade II-IV dysphagia, are presented in Fig. S1 of the supplementary material, which represents the graphical discrimination assessment. As shown in Table 2, the Brier scores also indicated that the accuracy of the original model was not as high as the other calibrated models in the validation cohort. Furthermore, the original model presented the highest difference between the predicted and calibrated probabilities according to the average absolute difference in predicted and calibrated probabilities (Eavg). The CTP selected the “revised model” (new predictor coefficients) as the ideal updated model after the likelihood ratio tests between the calibrated models (“re-calibration in the large”, “logistic recalibration”, “model revision”) against the “original model”. In addition, the Hosmer-Lemeshow test for the calibrated models showed non statistically significant p values (higher p-value for the “revised model”) which indicated that there was no evidence for a disagreement or difference between the predicted and observed NTCP values.

**Table 2:** Performance of the of the original NTCP and the calibrated models in the patient cohort we used (n = 277).

Models	Original NTCP model	Re-calibration in the large	Logistic recalibration	Model revision/update
Performance measure	Discrimination			
AUC (95 % CI) of the original NIPP model	0.82	–	–	–
AUC (95 % CI)	0.80(0.75–0.85)	0.80(0.75–0.85)	0.80(0.75–0.85)	0.83(0.78–0.88)
Sensitivity	0.71	0.76	0.78	0.80
Specificity	1	0.66	0.63	0.67

Calibration evaluation	Calibration			
Calibration intercept	0	1.11	1.41	–
Calibration slope	1	1	1.18	–
Brier	0.20	0.16	0.16	0.15
Emax	0.30	0.06	0.08	0.12
Eavg	0.16	0.02	0.02	0.03
E90	0.27	0.04	0.03	0.06
Hosmer–Lemeshow test of the original NIPP model	p = 0,93	–	–	–
Hosmer–Lemeshow test	x^2^ = 74.48,p value≪0,05	x^2^ = 6.68,p value = 0,57	x^2^ = 6.82,p value = 0,55	x^2^ = 1.87,p value = 0.98

Abbreviations: 95 % CI:confidence interval with a 95 % confidence level, AUC:the area under the receiver-operating characteristic curve, Brier: Brier score (average squared difference in predicted and actual probabilities), Emax/E90/Eavg: Maximum/90th quantile, average absolute difference in predicted and calibrated probabilities,x^2^ = chi-square statistic is a measure of the difference between the observed and expected frequencies of the outcomes of a set of events or variables.

The values of the different predictor coefficients of the “original” and the selected “revised model” by the CTP is presented in Table 3. The difference in the intercept values as well the tumour location and dysphagia scores of the models, potentially indicates the improvement of the calibration curve of the revised model compared to the original one.

**Table 3:** Intercept and coefficients of the original and revised model by the CTP.

Parameters	Original model	Revised model selected by the CTP
Intercept	−4.05	−6.99
Dmean Oral cavity coefficient	0.03	0.01
Dmean PCM superior coefficient	0.02	0.06
Dmean PCM medium coefficient	0.01	−0.01
Dmean PCM inferior coefficient	0.01	0.01
Tumour location coefficient	1	2.17
Baseline dysphagia score coefficient	1	−4.72

**Fig. 2:**
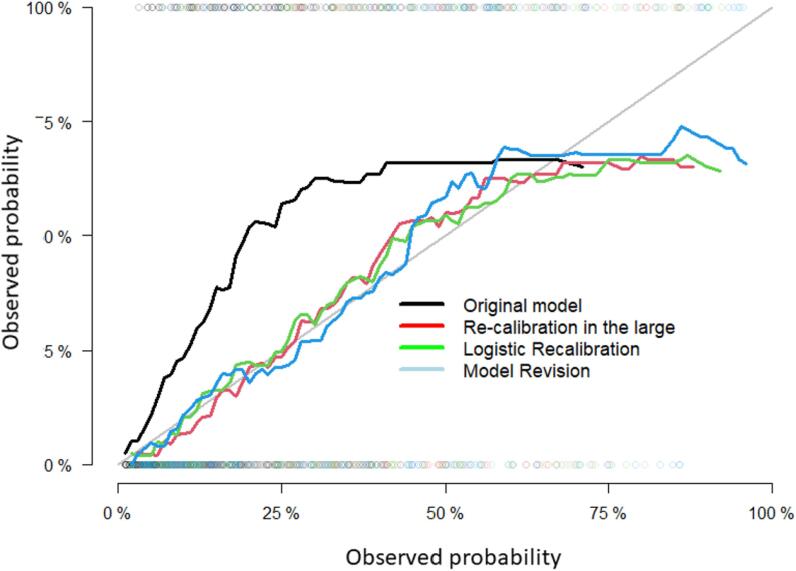
Calibration curves of the different NTCP grade II-IV six months dysphagia models as indicated by the CTP, i) original NTCP model, ii) re-calibration in the large, iii) Logistic recalibration iv) Model revision.

Fig. 2 shows that the original model underestimated the risk of grade II-IV dysphagia in the time-point of six months after the end of the RT treatment. Furthermore, the three calibration levels of the “re-calibration in the large”, “logistic recalibration” and “model revision” models, significantly improved the agreement between the predicted and observed NTCP risks. The individual calibration curves for each calibrated NTCP grade II-IV dysphagia model including the non-parametric estimate of the calibration relationship between the actual and predicted NTCP values can be found in the figures S2-S5 of the supplementary material.

## 4 Discussion

Several factors of model transferability and reproducibility can be taken into consideration for external validation studies such as geographical location (location of the hospital/patients) or methodological (RT treatment protocol used) transferability. However It is highly important to continuously update the models that may change over time. Therefore, our study successfully implemented an independent validation of a dysphagia NTCP model which has been externally validated already in two proton therapy centres and is used within the model-based selection of PT patients. Moreover, we examined whether the model needed an update when applied to the independent patient cohort.

The ideal scenario in the case of the external validation of a prediction model in an independent cohort includes its high performance in terms of statistical metrics such as sensitivity, specificity and the area under the ROC curve. According to Van Calster et al. [23] this high performance can be in other words called “strong calibration” and implies that a model is totally correct in the validation dataset. However, according to the same study, the “strong calibration” can be unrealistic in real-word data. Therefore, the external validation of NTCP models in independent cohorts may require a specific update mechanism that takes into account the different factors that make the external validation of NTCP models unsuccessful [24], [25].

In our study, there were minor differences in the calibration assessment (quantitative and graphical) of the three calibrated models. The Hosmer–Lemeshow test showed that there was no statistically significant difference between the distribution of the predicted and observed NTCP values(p values > 0.05), and therefore there was no evidence that the updated models did not “fit” well in the validation cohort we used. However, it is worth highlighting that the goodness of fit Hosmer–Lemeshow test is not proof that a model “fits” well in a cohort. This test indicates that there is enough evidence for the rejection of the hypothesis that a model is correctly specified [26]. Despite our initial goal to externally validate the NTCP dysphagia model using an independent patient cohort by assessing its transferability, there were some discrepancies between the methods used in this study and the methodologies proposed by other studies [23], [27]. Therefore some limitations should be taken into account. First, as stated by the NIPP [8]**,** in the validation datasets of the original NTCP model, missing values were computed using multiple imputation. In our case, we included only complete cases and did not perform any imputation method to account for missing values. This is possibly-one of the reasons that the original was not selected by the CTP and its performance was not as high as the revised model which was selected by the CTP. Secondly, according to Van Calster et al. [23], it is recommended that at least 200 events and 200 non-events were required for the development of flexible calibration curves. In our dataset consisting of 277 patients, we included 87 patients who developed grade II-IV dysphagia (events) in six months after RT and 190 patients who did not (non-events) for creating and assessing graphically the calibration plots of the different levels of calibrations according to the CTP. Moreover, according to Van de Bosch et al. [27], an external validation of the updated model was recommended in the case of a selection of the revised model by the CTP. In our case, the model selected by the CTP was not validated by another external and independent dataset and so is at risk of overfitting and over-optimistic performance. The aforementioned reported limitations of our study have to be taken into account in the case of a potential independent validation of the revised model by other external centres. Therefore, we encourage the independent external validation by other RT institutions (inter)nationally of the revised model selected by the CTP for its transferability and generalisability assessment.

Taking into account the potential effect of the dysphagia baseline scores as a predictor in the NTCP dysphagia models, according to Fig. 1 and the NIPP [8] there was a difference in the incidence of baseline dysphagia between the development cohort of the original NTCP model (25 %) and the external validation cohort we used (15 %). This difference could possibly have contributed to the selection of the “revised model” by the CTP in our case. However, it is worth mentioning that this difference of approximately 10 % can be explained by the chance of variations in the dysphagia Common Toxicity Criteria for Adverse Events version 4.0 (CTCAEv4.0)-physicians’ based scoring between the centre that developed the NTCP dysphagia model and the validation centre in the different timepoints. Furthermore this can be one of the possible reasons that explain the underestimation of the risk of patients to develop equal or greater than grade two dysphagia from the original model as shown in Fig. 2. Similar variations have been observed in previous external validation studies for head and neck cancer studies for the WHO performance status for instance [28].

Another factor that can influence the performance of a NTCP model containing dosimetric predictor OARs variables is the delineation method used for the OARs contours. We included patients with manual OARs delineations for the dosimetric OARs NTCP predictor variables. The last few years, several studies proposed the implementation of AI-based techniques for the automation of the delineation procedure for head and neck cancer patients [29], [30]. Interobserver variability among different clinicians for head and neck patients was a common phenomenon [31] that can impact the quality of dosimetric data included in a prediction model and therefore the performance of it in different independent patients’ cohorts.

The need for external validation of NTCP models was stressed by the Danish study of Pedersen et al. [32]. This study examined dosimetric photon and proton based NTCP parameters differences by internally validating the NTCP model of Lyman-Kutcher-Burman (LKB) using prospective treatment and morbidity data of PT treated prostate cancer patients. The authors highlighted the importance of NTCP models update and external validation due to clinical practice patterns changes as they concluded that dosimetric parameters such as the mean dose to 50 % of the target volume (D50) was different from the typical photon-based LKB NTCP model.

As a next step, we aim to implement federated learning techniques adhering to the FAIR principles [11]using the Personal Health Train (PHT) infrastructure [12] by exchanging statistical algorithms. Those algorithms can use the CTP approach in a privacy-preserving manner (ie. without the exchange of patient data; only statistical results). Transforming the different data items in a machine readable FAIR format across the different participated proton therapy centres we aim to include larger patients’ cohorts for the development and validation of the NTCP models including patients who are treated with different RT treatment protocols for head and neck cancer.

In conclusion, with this study we performed an independent validation of the NTCP grade II-IV dysphagia model (primary setting) which is used for the selection of patients for PT. We concluded that the performance of the model in an independent and external patients’ cohort was good. There was still room for improvement, however, as the distribution of the observed compared to the predicted probabilities of the model according to the calibration plot generated was not ideal. Following the CTP methodology, it was indicated that the model should be updated and calibrated. We therefore, based on the CTP, selected the revised version of the “original model” with updated intercept and predictor coefficients for further development. The revised version of the model had a high discrimination in the independent validation cohort, but an additional external and independent validation from other RT centres is needed to further evaluate its robustness and transferability.

## Declaration of Competing Interest

The authors declare the following financial interests/personal relationships which may be considered as potential competing interests: Prof. Dr. Andre Dekker and Dr. Johan Van Soest are founders and stock owners of Medical Data Works B.V. which has products that are related to knowledge graphs. Prof. Dr. Johannes Langedijk has a research agreement between the Department of Radiation Oncology, University of Groningen, University Medical Centre Groningen. The Netherlands and the companies IBA and RaySearch. Furthermore, Prof. Dr. Johannes Langedijk is a member of the Global Advisory Board of the company IBA for the research and development of the company. Moreover, Prof. Dr. Johannes Langedijk is a member of the RayCare Clinical Advisory Board of the company RaySearch as he provides advices on the development of RayCare. Dr. Rianne Fijten has received research funding from Varian Medical Systems. In addition, she is the chair of the Open Science Community Maastricht and a member of the Dutch Open Science Communities NL (OSC-NL) steering committee.
