# Supplementary material for: Independent validation of a dysphagia dose response model for the selection of head and neck cancer patients to proton therapy
Source: Phys Imaging Radiat Oncol. 2022 Sep 17;24:47–52. doi: 10.1016/j.phro.2022.09.005 (PMC9493379; doi:10.1016/j.phro.2022.09.005)
Supplement: Supplementary data 1 [file mmc1.docx]

**Supplementary material**

| **Table S1 NTCP development cohort characteristics** | |
| --- | --- |
| **Demographic characteristics** | |
| **Gender** | **N(%)** |
| Male | 560(75) |
| Female | 190(25) |
| **Clinical characteristics** | |
| **Tumour location** | **N(%)** |
| Oral cavity | 44(6) |
| Oropharynx | 271(36) |
| Nasopharynx | 30(4) |
| Hypopharynx | 71 (10) |
| Larynx | 334(44) |
| **T-stage** | **N(%)** |
| Tis-T2 | 363(48) |
| T3-4 | 387(52) |
| **N-stage** | **N(%)** |
| N0 | 333(44) |
| N+ | 417(56) |
| **Dosimetric characteristics-predictors of the NTCP model for dysphagia grade ≥ 2 at 6 months (Gy)** | |
| Dmean Oral cavity | 43.9 |
| Dmean PCM superior | 52.3 |
| Dmean PCM medium | 55.7 |
| Dmean PCM inferior | 52.3 |

| **Table S2:Definition of the different models according to the closed testing procedure (CTP)** | |
| --- | --- |
| **Definition** | **Estimated parameters** |
| Original NTCP grade II-IV dysphagia model. | No parameters |
| Re-calibration in the large | Intercept |
| Logistic Recalibration | Intercept and slope |
| Model revision/update | Logistic regression coefficients |


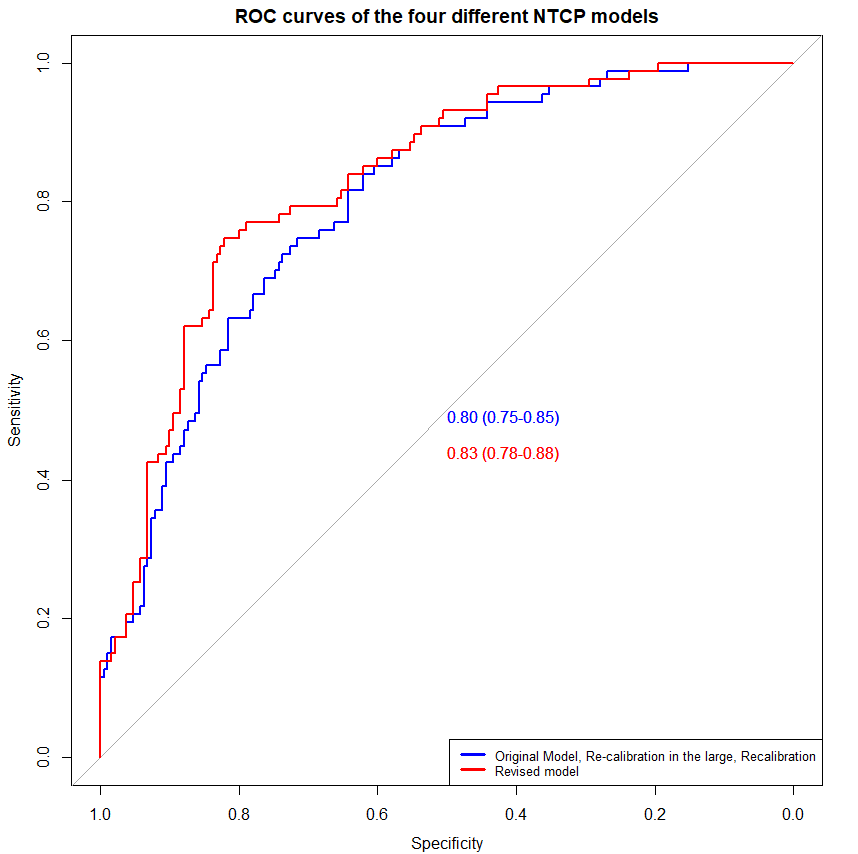


**Figure S1**: ROC curves of four different models indicated by the CTP. The revised model selected by the CTP, showing good discriminative performance in MAASTRO’s cohort as indicated by the AUC values (>0.75). The re-calibration of a prediction model has no influence on an ROC curve as the cut-off points might change, but the confusion matrices remains the same. Therefore, The AUC values for the original, recalibrated in the large and recalibrated model, remain the same (blue colour line). Abbreviations: ROC = receiver operating characteristic; NTCP = normal-tissue complication probability; AUC = area under the curve.

For the creation of the calibration curves of the different models presented in table 2 of the main text of the study we used the package “val.prob” of the RStudio library “rms”[^1^](https://www.zotero.org/google-docs/?B3zLCf). In the different figures, the dashed curve represents the non-parametic estimate of the calibration probabilities between the predicted and observed/actual values. The grey diagonal line presents the ideal probability distribution (intercept=0 and slope=1).

**
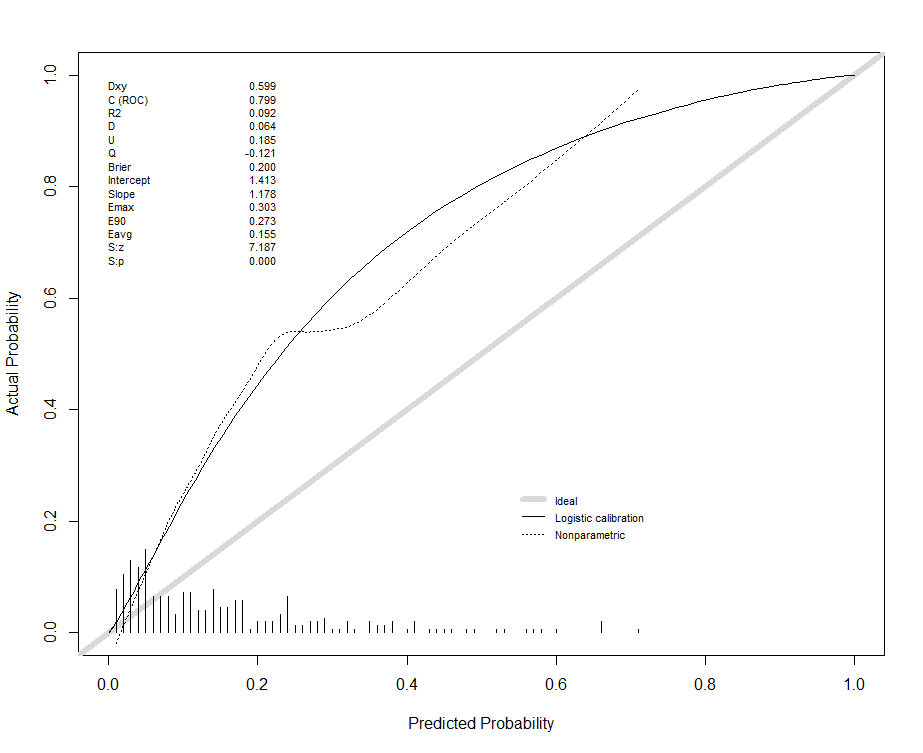
**

**Figure S2:** The calibration curve of the original NIPP[^2^](https://www.zotero.org/google-docs/?7turUi) NTCP grade ≥ 2 dysphagia model. As it is shown according to the distribution of the predicted and actual probabilities, the original model underestimates the risk of the head and neck MAASTRO patients (N=277) to develop grade ≥ 2 dysphagia six months after the end of the RT (calibration curve above the diagonal line). **Abbreviations:** Dxy: Somer’s rank correlation, C(ROC): Area Under the Curve for discrimination assessment, R2: Nagelkerke-Cox-Snell-Maddala-Magee R-squared index, D: discrimination index, U: unreliability index, Q: quality index, Brier: Brier score (average squared difference in predicted and actual probabilities), Emax/E90/Eavg: Maximum/90th quantile, average absolute difference in predicted and smoothed calibrated probabilities, S:z/S:p the z and two sided p-value of the Spiegelhalter test for calibration accuracy.

**
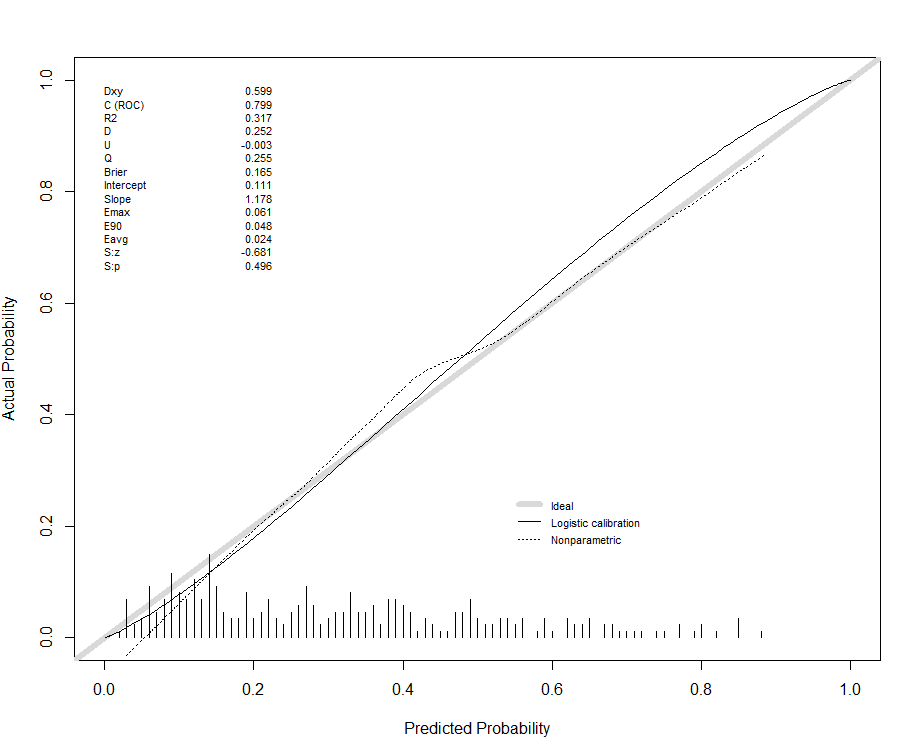
**

**Figure S3:** The calibration curve of the “Re-calibration in the large” (estimation of an updated intercept of the original model) . As it is shown, there is an improvement of the distribution of the predicted and actual probabilities indicated by the brier score and the average absolute difference in predicted and smoothed calibrated probabilities (Eavg). **Abbreviations:** Dxy: Somer’s rank correlation, C(ROC): Area Under the Curve for discrimination assessment, R2: Nagelkerke-Cox-Snell-Maddala-Magee R-squared index, D: discrimination index, U: unreliability index, Q: quality index, Brier: Brier score (average squared difference in predicted and actual probabilities), Emax/E90/Eavg: Maximum/90th quantile, average absolute difference in predicted and smoothed calibrated probabilities, S:z/S:p the z and two sided p-value of the Spiegelhalter test for calibration accuracy.


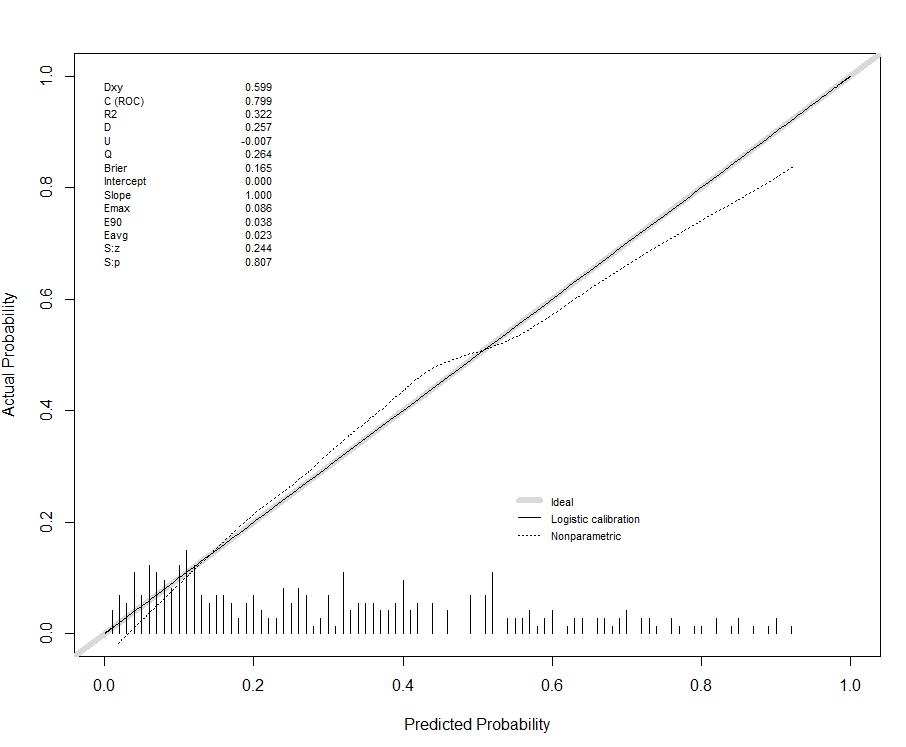


**Figure S4:** The calibration curve of the “Logistic recalibration” model (estimation of an updated intercept and slope of the original model) . As it is shown, there is an improvement of the distribution of the predicted and actual probabilities indicated by the brier score and the average absolute difference in predicted and smoothed calibrated probabilities (Eavg) **Abbreviations:** Dxy: Somer’s rank correlation, C(ROC): Area Under the Curve for discrimination assessment, R2: Nagelkerke-Cox-Snell-Maddala-Magee R-squared index, D: discrimination index, U: unreliability index, Q: quality index, Brier: Brier score (average squared difference in predicted and actual probabilities), Emax/E90/Eavg: Maximum/90th quantile, average absolute difference in predicted and smoothed calibrated probabilities, S:z/S:p the z and two sided p-value of the Spiegelhalter test for calibration accuracy.


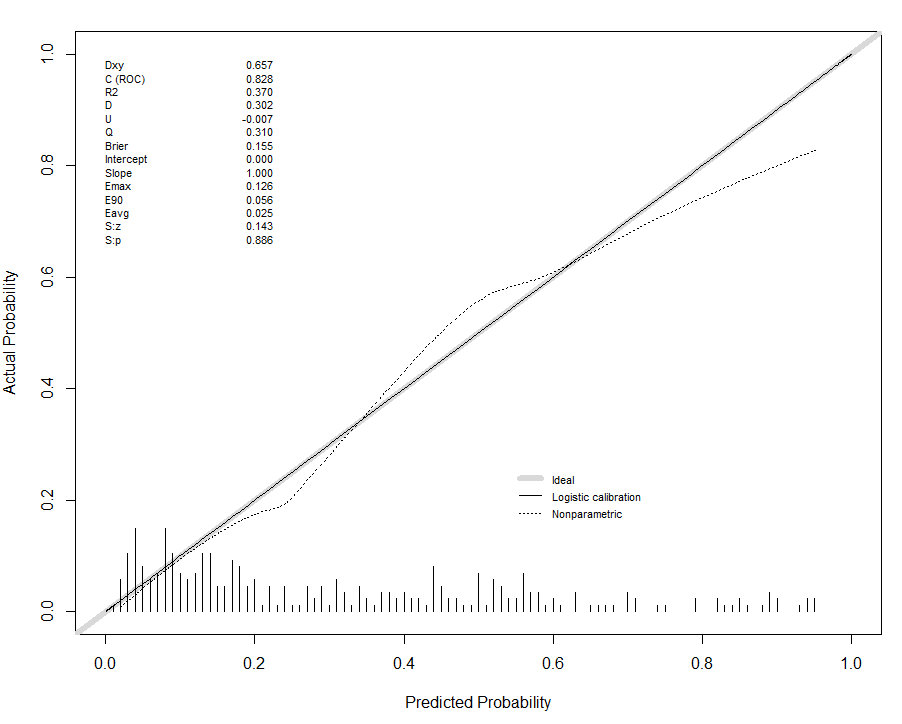


**Figure S5:** The calibration curve of the “Revised model” (estimation of an updated intercept and slope of the original model) . As it is shown, there is an improvement of the distribution of the predicted and actual probabilities indicated by the brier score and the C(ROC). **Abbreviations:** Dxy: Somer’s rank correlation, C(ROC): Area Under the Curve for discrimination assessment, R2: Nagelkerke-Cox-Snell-Maddala-Magee R-squared index, D: discrimination index, U: unreliability index, Q: quality index, Brier: Brier score (average squared difference in predicted and actual probabilities), Emax/E90/Eavg: Maximum/90th quantile, average absolute difference in predicted and smoothed calibrated probabilities, S:z/S:p the z and two sided p-value of the Spiegelhalter test for calibration accuracy.
